# Study of the Usability of Spaced Retrieval Exercise Using Mobile Devices for Alzheimer’s Disease Rehabilitation

**DOI:** 10.2196/mhealth.3136

**Published:** 2014-08-14

**Authors:** Ahmad Zmily, Yaser Mowafi, Ehab Mashal

**Affiliations:** ^1^School of Computer Engineering and Information TechnologyGerman Jordanian UniversityAmmanJordan; ^2^Darat Samir ShammaShafa BadranJordan

**Keywords:** Alzheimer disease, Alzheimer disease rehabilitation, spaced retrieval exercise, usability study, mobile human computer interaction

## Abstract

**Background:**

Alzheimer's disease (AD) is an irreversible brain disease that slowly destroys memory and thinking skills, and eventually the ability to carry out the simplest daily tasks. Recent studies showed that people with AD might actually benefit from physical exercises and rehabilitation processes. Studies show that rehabilitation would also add value in making the day for an individual with AD a little less foggy, frustrating, isolated, and stressful for as long as possible.

**Objective:**

The focus of our work was to explore the use of modern mobile technology to enable people with AD to improve their abilities to perform activities of daily living, and hence to promote independence and participation in social activities. Our work also aimed at reducing the burden on caregivers by increasing the AD patients’ sense of competence and ability to handle behavior problems.

**Methods:**

We developed ADcope, an integrated app that includes several modules that targeted individuals with AD, using mobile devices. We have developed two different user interfaces: text-based and graphic-based. To evaluate the usability of the app, 10 participants with early stages of AD were asked to run the two user interfaces of the spaced retrieval memory exercise using a tablet mobile device.

**Results:**

We selected 10 participants with early stages of AD (average age: 75 years; 6/10, 60% males, 4/10, 40% females). The average elapsed time per question between the text-based task (14.04 seconds) and the graphic-based task (12.89 seconds) was significantly different (*P*=.047). There was also a significant difference (*P*<.001) between the average correct answer score between the text-based task (7.60/10) and the graphic-based task (8.30/10), and between the text-based task (31.50/100) and the graphic-based task (27.20/100; *P*<.001). Correlation analysis for the graphic-based task showed that the average elapsed time per question and the workload score were negatively correlated (−.93, and −.79, respectively) to the participants’ performance (*P*<.001 and *P*=.006, respectively).

**Conclusions:**

We found that people with early stages of AD used mobile devices successfully without any prior experience in using such devices. Participants’ measured workload scores were low and posttask satisfaction in fulfilling the required task was conceivable. Results indicate better performance, less workload, and better response time for the graphic-based task compared with the text-based task.

##  Introduction

### Background

Alzheimer's disease (AD) is an irreversible brain disease that slowly destroys memory and thinking skills, and eventually the ability to carry out the simplest daily tasks. Currently, there is no cure for the disease, which worsens as it progresses, and eventually leads to death. The Alzheimer's Association estimates that 1 in 8 older Americans is living with AD totaling approximately 5 million Americans [1].

AD is the most common cause of dementia among older people. Dementia is the loss of cognitive functioning including thinking, remembering, and reasoning and the loss of behavioral abilities, to such an extent that it interferes with a person's daily life and activities. Dementia ranges in severity from the mildest stage, when it is just beginning to affect a person's functioning, to the most severe stage, when the person must depend completely on others for basic activities of daily living [2].

Health care professionals have tended to overlook the needs and the requirements of Alzheimer's individuals for physical exercises and activity programs. Because of the memory retention problems, many health care professionals feel that the majority of persons with AD have little, if any, rehabilitation potential. Besides retention problems, many clinicians also take the "they're just going to get worse anyway" approach in their clinical decision making [3].

Recent studies [3] have shown that people with AD may actually benefit from physical exercises and rehabilitation processes. Studies showed that rehabilitation would also add value in making the day for an individual with AD a little less foggy, frustrating, isolated, and stressful for as long as possible. In addition, rehabilitation helps the individual with AD on living as fully as possible with whatever time he has. In general, rehabilitation is about the quality of life; it adds life to the years, not more years to the life.

For individuals with AD, rehabilitation should focus on people's abilities, rather than their disability. The goal is to maintain as high a quality of life as possible for as long as possible [3]. This may help to enhance people's ability to take part in meaningful activities within their environment and enjoy family events.

In recent years, mobile devices have improved rapidly in processing power, embedded sensors, storage capacity, and network data rates. Mobile devices today have evolved from merely being phones or simple personal digital assistances to full-fledged computing, sensing, and communication devices. These advances in mobile device technology have paved the way for exciting new apps. Mobile devices can be used as medical devices for measuring blood pressure, measuring glucose levels, performing portable ultrasounds, and even testing for sexually transmitted diseases.

In previous research [4], we have focused on developing ADcope, an integrated app that includes several modules that target individuals with AD, using mobile devices. The goal for ADcope is to maximize the patients' remaining capabilities and avoid excess disability, improving their overall quality of life. In this paper, we focus on the usability aspects of ADcope. Specifically, we studied the usability of the Spaced Retrieval Exercise module on a group of people who are diagnosed with early stage AD.

### Approach

AD is currently an incurable disease and worsens progressively. Our approach in dealing with this disease focuses on two aspects: finding ways to help the patient cope with the disease, and slowing down the pace of decline in quality of life as the disease worsens. A combination of advanced mobile devices equipped with near field communication (NFC) and NFC tags will be used to meet the challenges of the two focus areas. Mobile devices running the Google Android operating system (OS) have been selected because currently, many mobile devices supporting NFC are running the Android OS.

### General Requirements in Dealing With AD Patients

Our approach was based on general standards recommended when dealing with people with dementia [3]. This includes: (1) using simple language when interacting with AD patients; (2) repeating instructions several times; (3) instructions should be broken into simple steps and given one at a time; (4) allow the AD patient ample of time to respond or react; and (5) all messages should be as short as possible.

### Helping the Patient Cope With the Disease

**Figure 1:**
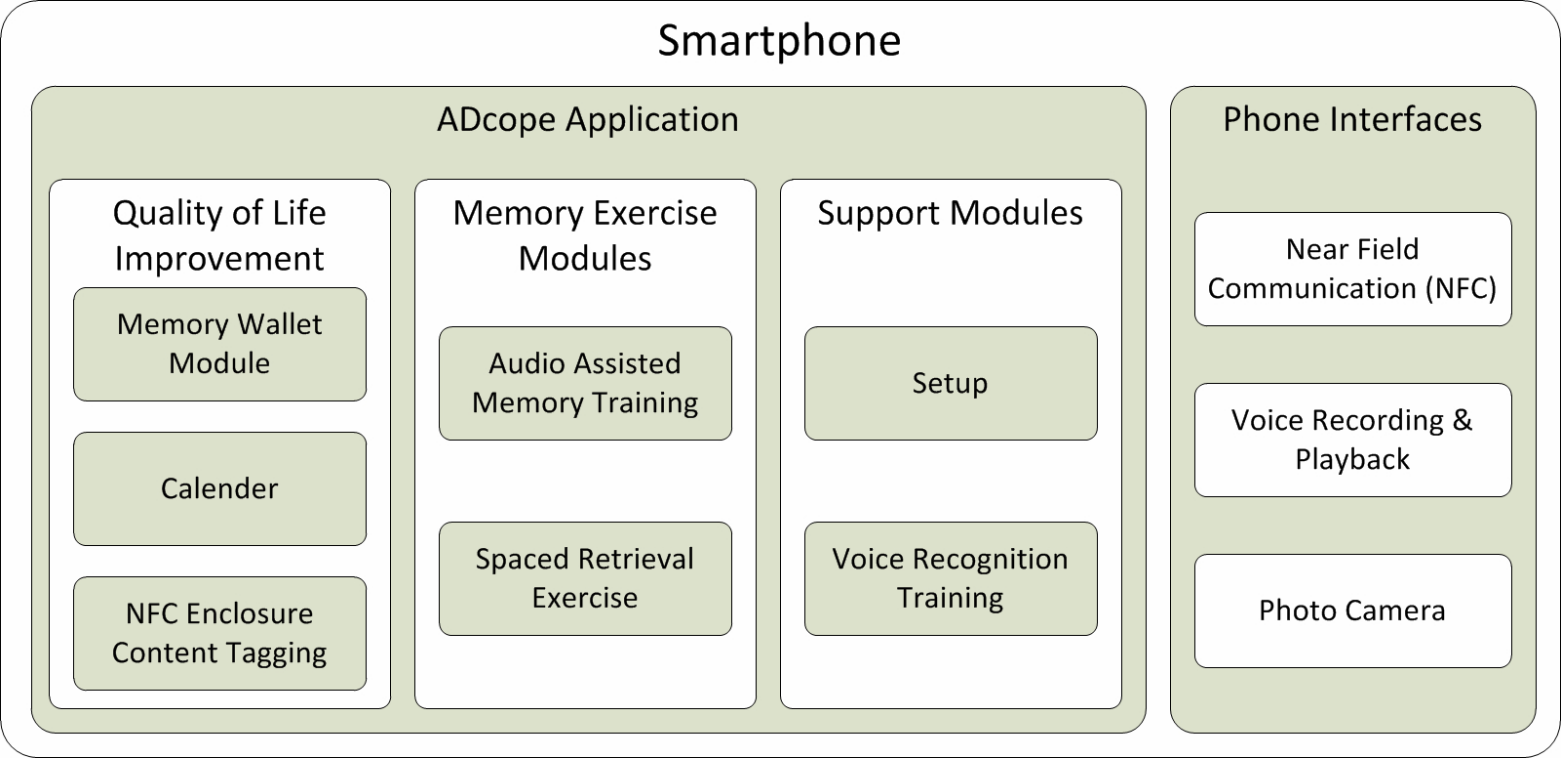
ADcope modules and interfaces.

Since AD is not a curable disease, our approach focused on maintaining the highest possible quality of life of AD patients. An app, named ADcope, with several modules was developed on mobile devices to support this approach [4]. Figure 1 is a block diagram of the app showing the modules and mobile device interfaces. The app and its modules were designed to help the patient in many aspects of the daily life. The continuous interaction of the patient with the mobile device as he moves through his daily life ensures that the patient does not forget about using the mobile device. In addition, ADcope provides an option to remind the AD patient to use the mobile device.

The first module is a memory wallet, as suggested by Bourgois et al [5]. They suggested the use of a wallet that contained 30 pictures and sentences about familiar persons, places, and events. The patients managed to learn to use the wallet to improve their conversations by making more accurate factual statements. The module allows the user to take photos of the familiar people, places, and events. The patient is then given a chance to tag the photos with phrases that reminds him of the subject of the photo. Photos of people can also be tagged with voice samples of the person in the photo. The patient can go back to the wallet as frequently as needed to be reminded of these people, places, and events. The memory wallet also use the tagged voice samples to automatically play back the photo and tagged phrases anytime the voices in the background match the tagged voice samples helping the patient to refer to the wallet to instantly get help on recognizing the people around him without having to sift through the wallet.

The second module is a calendar with reminders of all daily activities that need to be performed. The events can refer to information in the memory wallet such as photos to help the patient recognize the person he needs to talk to or the place he needs to go. The calendar also includes the recurring event to review the memory wallet.

The third module uses NFC tags that are placed on various things, including drawers and doors. As the person touches these with the mobile device, the mobile device displays a list of content of the drawer or room. This saves the person from having to open them for inspection when looking for something. This can also be used by a new caretaker to facilitate fetching items requested or needed by the patient since they may not have prior knowledge of how things are arranged.

### Exercising the Patients’ Memory

Although AD is an incurable disease, some memory exercises may help in retaining critical information longer. Researchers have identified several exercises and training techniques that patients with dementia should perform periodically. The exercises include Audio Assisted Memory Training as identified by Arkin [6] and Spaced Retrieval, which was first described by Landauer and Bjork in 1978 [7]. In Audio Assisted Memory Training an audiocassette recorder was used to playback narratives of biographical information then interactive quizzes are performed to exercise the retrieval process. This technique has resulted in substantial learning with most patients. The Spaced Retrieval exercises are based on asking a questions and requiring an immediate response. The questions are then repeatedly asked with the time between each repetition systematically lengthened until the patient demonstrates the ability to recall information in everyday life. This technique has been increasingly used with AD patients to teach important information and skills needed to improve daily life.

These exercises have been integrated into the ADcope app as separate modules. The Audio Assisted Memory Training module replicates the audiocassette recorder approach by playing back audio files of the biographical information and quizzing the patient regarding the information. The targeted answers are very short and mostly consist of one or two words. The simplicity of the answers is critical for successful exercises based on the general recommendations when dealing with AD patients. The simplicity of the answers also enables the use of voice recognition techniques to automatically validate correctness of responses.

The Spaced Retrieval exercise module has been designed to execute in two phases: an assessment phase and a training phase. During the assessment phase, the current memory recall ability is assessed by presenting a piece of information, and then the patient is quizzed about it at a later time. If the recall fails, the information is reiterated, and quiz repeated again later. The length of time between quizzes is reduced until memory recall is successful. The final duration between information presentation and successful recall is stored as the basis for initial duration in future training. The second phase is the training phase in which the module follows the Spaced Retrieval recommendations for training recall of information. The module starts with the duration of time between information presentation and quizzing as determined in the first phase and the length of time between information presentation and quizzing is lengthened with every successful retrieval and maintained with every failure.

### Supporting Modules

The modules described in the previous sections require substantial setup and training. The ADcope app includes a setup module, which will guide the caregiver (or patient if he is able to) through all of the setup steps, which is outlined in Textbox 1.

The app needs to be able to recognize voices and match voice replies with correct answers. The ADcope app includes a separate module for training the voice recognizer. The module displays words and asks the patient to read them. A spectral analysis is then done on the words and stored for future voice recognition.

**ADcope setup module.**

- Recording audio files of biographical data: the user is asked to narrate the desired biographical phrases and signal the termination of recording. The user can record several audio files that are randomly played back in the Audio Assisted Memory Training module.
- Recording of biographical quiz questions and answers: the user is repeatedly asked for questions that are recorded. After each question, the user is asked to type in the correct answer. The answers are currently limited to numbers (such as age, year, and quantities) or single words due to the limitation of the voice recognition libraries used in the development of the app.
- Spaced retrieval exercises: the ADcope app has some built-in spaced retrieval exercises based on general knowledge facts. However, the setup module allows the user to add additional fact-question pairs. The user also has the option to setup the Spaced Retrieval module to exclusively use the user-added facts.
- Memory Wallet initialization: the user is asked to take photos of people, places and events and type in a word or a phrase for each one. The user can also optionally tag the photo with a voice sample of the person in the photo. The user is allowed to defer this part of the setup to later and build the memory wallet as he meets people or visits places.
- NFC tagging: The user is asked to enter a note that describes the item being tagged. For example, this could be contents of drawers. The user is then asked to place the smartphone next to the tag and the ADcope app uses the internal NFC writer of the smartphone to write the information to the tag.
- Calendar: In the last step of the setup procedure. The user is asked to enter recurring calendar event with optional photos for each event.

## Methods

### Study Scope

In this study, we evaluate the usability of the proposed ADcope app. Specifically our main objective is to evaluate the usability of the Spaced Retrieval exercise. The following section describes in details the selected participants, apparatus, study procedure, and the collected measures.

### Participants

The participants considered in the study were volunteers from Darat Samir Shamma, a senior residential care facility and housing located in Amman, Jordan. The participants were primarily seniors, with early stages of AD. It is important to note that finding participants with early stages of AD was very challenging. Ten participants were asked to perform the Spaced Retrieval exercise on a tablet device while sitting in a room (NCT02005380). The mean age of the participants was 75 years and the SD was 9.6. Basic demographic characteristics were recorded for each participant. Table 1 summarizes the demographic factors for the participants’ age, mobile device ownership, mobile device usage frequency, and familiarity with tablets along with their current ability of using mobile devices.

As shown in Table 1, most of the participants in this study were not frequent handheld mobile devices users and had no familiarity with tablets. However, the experimental tasks performed on the tablet device were designed in which prior experience with handheld mobile devices or tablets was not required. Additionally, all participants were initially acquainted and instructed on performing the experiment goals and tasks. Tasks were designed with minimal input needed to accomplish the exercise. Each participant had been assigned to perform the same set of tasks on a tablet device while sitting at a table.

**Table 1:** Participants' demographics summary (N=10).

Characteristics		
**Age, years**		
	Average	75.1
	SD	9.6
**Gender, n (%)**		
	Male	6 (60)
	Female	4 (40)
**Handheld mobile device owner? n (%)**		
	Current	7 (70)
	Never	3 (30)
**Handheld mobile device use frequency, n (%)**		
	>1/day	4 (40)
	1/day	3 (30)
	<1/day	3 (30)
**Familiarity with tablets, n (%)**		
	Yes	3 (30)
	No	7 (70)

### Apparatus

Seven-inch Samsung Galaxy Tab 2 devices running Android OS were used in this study. The app was developed using the Android software development kit (SDK). The app kept a log of participants’ response times, total time of each trial, and the total time for each participant to complete his/her overall task. At the beginning of each experiment, participants were briefed about the goal of the study and instructions on how to use the device and run the app to complete the required task. Upon completion of the trials, participants were requested to fill in a posttask questionnaire to rate the performed task degree of difficulty along with the National Aeronautics and Space Administration task load index (NASA-TLX) workload assessment [8].

NASA-TLX [8] is a subjective workload assessment tool that allows users to perform subjective workload assessments on operators working with various human-machine systems. NASA-TLX is a multidimensional rating procedure that derives an overall workload score based on a weighted average of ratings on six subscales. These subscales include mental demands, physical demands, temporal demands, own performance, effort, and frustration.

### Procedure

As described in the Approach Section, the Spaced Retrieval exercise has been designed to execute in two phases; an assessment phase and a training phase. During the assessment phase, the current memory recall ability is assessed by presenting a piece of information, and then the patient is quizzed about it at a later time. If the recall fails, the information is reiterated, and the quiz is repeated again later. The length of time between quizzes is reduced until memory recall is successful. The final duration between information presentation and successful recall is stored as the basis for initial duration in future training.

In the assessment phase of our developed spaced retrieval exercise, one sentence piece of information along with a text button at the bottom of the screen labeled “tap the screen to continue” was displayed on the tablet’s screen. Participants were instructed not to move onto the question until they felt they had known the content of the presented information. When the participant taps on the screen, the sentence disappears and a question with four choices related to the previously presented sentence appears on the screen after some delay. The delay was initially set to 90 seconds at the first trial. Once the participant taps on the screen, s/he was not allowed to go back to the passage. If the participant taps on the correct answer, the time space between the presented information and the related question for the next trial was increased by 10 seconds. If the participant taps on the wrong answer or fails to answer within 60 seconds, the same information was represented again and the time space was decreased by 10 seconds for the next trial. The assessment phase was limited to 10 different questions or 30 minutes of time. The delay time between the presented piece information and the display of the question for the last trial was used for the training phase of the program and was referred to as the recall time for the participant.

The second phase was the training phase, in which the same procedure as the assessment phase was repeated where one sentence piece of information was presented followed by a question with four different choices. The recall time logged at the end of the assessment phase is used for the duration of time between information presentation and quizzing. The length of time between information presentation and quizzing is lengthened with every successful answer and maintained with every wrong answer. Once the participant selects a correct answer, a feedback appears for 5 seconds on the screen, which read "correct answer" before moving to the next question. If the selected answer was incorrect a "wrong answer" feedback appeared on the screen for 5 seconds before repeating the same information for the failed trial. The training phase for our experiment was limited to 10 questions. Upon successfully completing all of the trials, a feedback appears at the bottom of the page, which read "well done, task completed."

The ADcope Spaced Retrieval exercise offers two types of quizzes: text-based and graphic-based scenarios. In this study, participants performed both of the two scenarios.

### Task 1: Text-Based Spaced Retrieval

**Figure 2:**
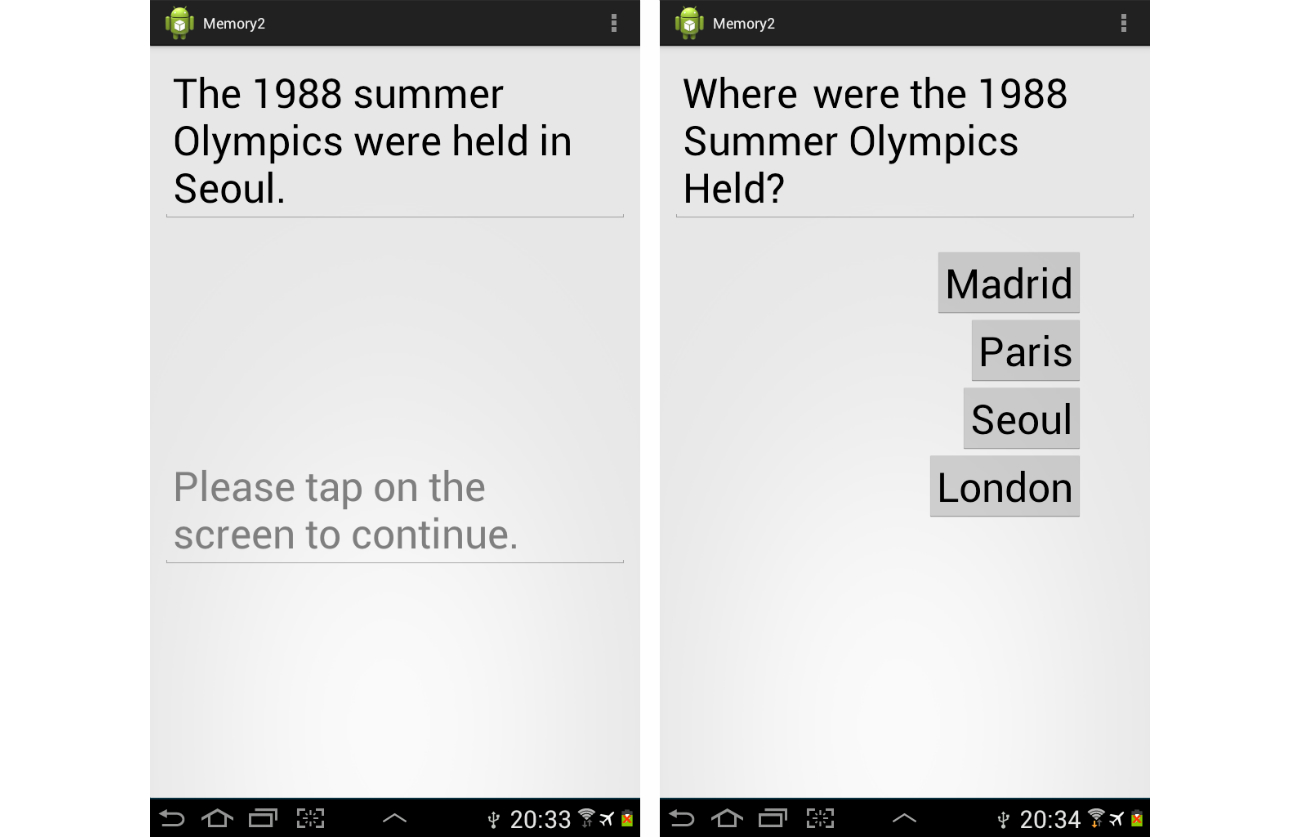
Screenshots of the text-based spaced retrieval exercise.

In this scenario, a one sentence piece of information was displayed on the tablet’s screen. The sentence was composed of 5 to 9 long words. After a delay, a question related to the previously presented sentence appears on the screen with four choices. Each of the answer choices consists of 1 to 2 words. The questions were general knowledge questions in sports, history, geography, and movies. Both of the assessment phase and training phase consist of 10 different questions each. The same presented sentences' order along with the same multiple choices were used for all participants. A screenshot of one of the presented information and the multiple-choice question screens are shown in Figure 2.

As some of the participants in our study have difficulties in reading text, we have used text to speech Android SDK to read the information, the questions, and the associated multiple-choices aloud. The developed app also supports two languages: English and Arabic. Participants were instructed to use their preferred language.

### Task 2: Graphic-Based Spaced Retrieval

**Figure 3:**
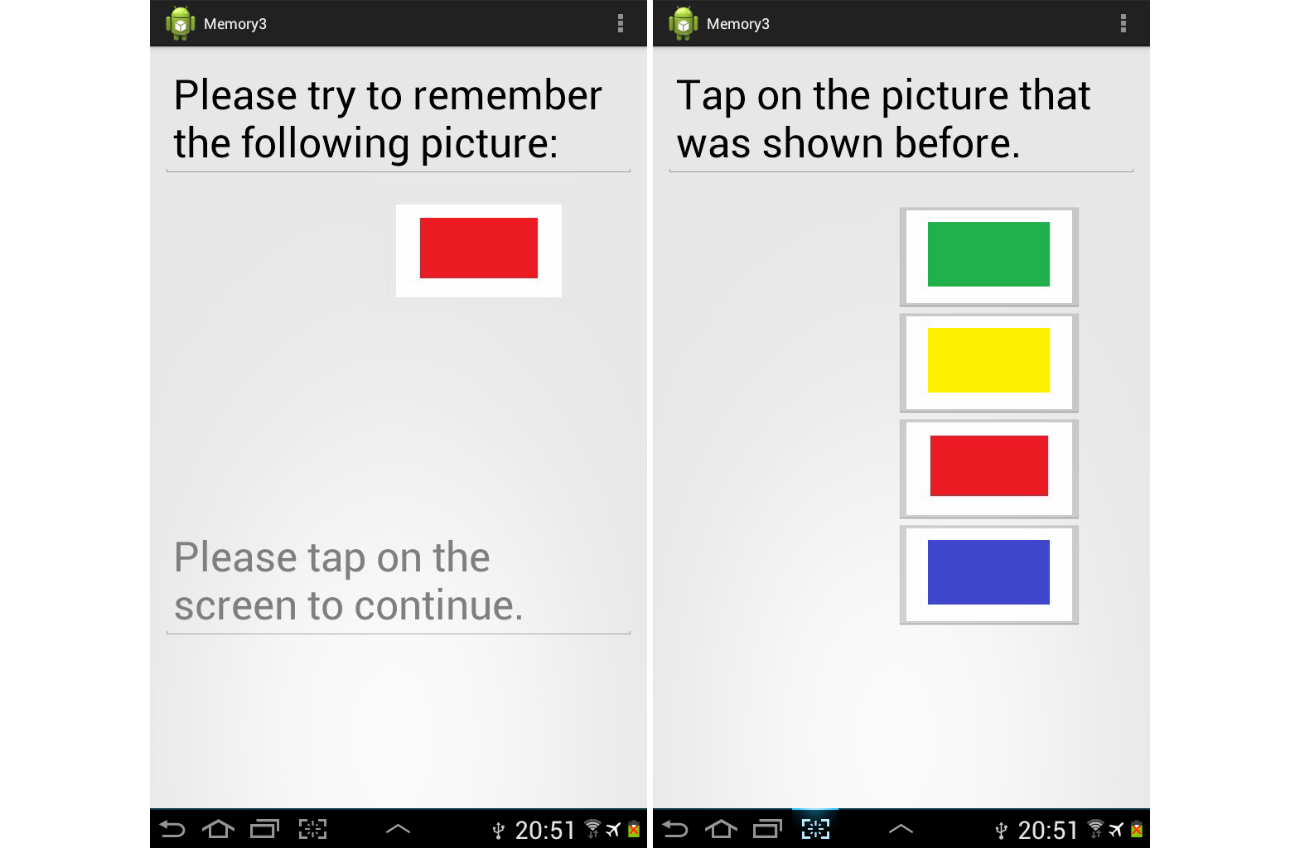
Screenshots of the graphic-based spaced retrieval exercise.

In this scenario, a shape or image was displayed on the tablet’s screen followed by a four multiple-choice images of which one matched the earlier presented image. The questions were created using simple geometrical shapes, flags, and traffic signs. The same 10 presented images order along with the 40 multiple choices were used for all participants. A screenshot of one of the presented information and the multiple-choice question screens are shown in Figure 3.

Experimental Measures

The recorded measures during the experiments are presented in Table 2. The measures are divided into objective and subjective measures and presented in Textbox 2.

**Table 2:** Collected measures.

Measure	Description
Average response time	The elapsed time between the question presentation and the participant selection of one of the answer choices.
Average elapsed time per question	The average elapsed time for answering presented question, excluding the recall delay time.
Correct answer score	Percentage of questions answered correctly from the first trial.
Posttask questionnaire score	Participants’ feedback related to his/her overall satisfaction in fulfilling the required task (between 0 and 7).
NASA-TLX^a^ score	The overall NASA-TLX subjective workload score (between 0 and 100).

^a^National Aeronautics and Space Administration task load index.

**Objective and subjective experimental measures.**

The objective measures are:

- The average response time: the elapsed time between the question presentation and the participant selection of one of the answer choices. It measures the average time it takes the participant to comprehend the question and select one of the four multiple-choice answers.
- Average elapsed time per question: the average elapsed time for answering presented question excluding the recall delay time. This measures the average time it takes the participant to successfully answer the question correctly excluding the delay between information presentation and quizzing.

Correct answer score: measures the percentage of questions answered correctly by the participants from the first trial.

The subjective measures are:

- Post task questionnaire score: measure the participants’ feedback related to his/her overall satisfaction in fulfilling the required task.
- NASA-TLX: measures the overall participants’ workload score in fulfilling the required task.

## Results

Table 3 presents the mean, minimum, maximum, and standard deviation for the average response time, average elapsed time per question, correct answer score, posttask questionnaire score, and NASA-TLX score for both the text-based and the graphic-based exercises. The mean average response time and the mean average elapsed time per question for the text-based task are higher than the graphic-based task (14.04 and 53.81 vs 12.89 and 34.84 seconds, respectively). Participants also scored higher correct answers with the graphic-based task compared with the text-based task (8.30/10.00 and 7.60/10.00, respectively). The mean values for the posttask questionnaire score for both of the text-based and graphic-based tasks were high (5.8/7.00 and 6.00/7.00, respectively) indicating that participants were satisfied in completing their two tasks successfully. The mean values for the NASA-TLX scores for both the text-based and graphic-based are relatively low (31.5/100 and 27.2/100, respectively) indicating that the two tasks require low workload.

To investigate the between-groups (text-based and graphic-based) difference, analysis of variance (ANOVA) test was conducted (Table 3). The results indicate significant differences between text-based and graphic-based tasks for average elapsed time per question (*P*=.047) and posttask questionnaire score (*P*=.02). The results also indicate significant differences between text-based and graphic-based tasks for average response time (*P*=.001), correct answer score (*P*<.001), and NASA-TLX (*P*<.001).

**Table 3:** Results compariosn between text- and graphic-based experiements.

Measure		Mean	Minimum	Maximum	SD	*F*	*P*
**Average response time**						23.94	.001
	Text-based	14.04	6.60	20.50	4.98		
	Graphic-based	12.89	5.90	19.50	4.49		
**Average elapsed time per question**						5.48	.047
	Text-based	53.81	9.30	186.00	54.67		
	Graphic-based	34.84	9.30	82.50	25.21		
**Correct answer score**						26.87	<.001
	Text-based	7.60	4.00	10.00	2.32		
	Graphic-based	8.30	6.00	10.00	1.70		
**Posttask questionnaire score**						7.69	.02
	Text-based	5.80	4.50	7.00	0.89		
	Graphic-based	6.00	4.50	7.00	1.03		
**NASA-TLX** ^a^						104.44	<.001
	Text-based	31.50	10.00	48.00	14.77		
	Graphic-based	27.20	10.00	40.00	10.64		

^a^National Aeronautics and Space Administration task load index

In order to investigate the relationship between the experimental measures and the participants’ performance in fulfilling their required task successfully, correlation analyses were run among all pairs of the experimental measures. The correlation results are shown in Table 4 for the text- and graphic-based tasks. The correlation between the posttask questionnaire and the participants’ performance in fulfilling the required task successfully did not identify any significance. For the text-based task, the average response time is negatively correlated (−.84) to participants’ performance and significant (*P*=.002). Similarly, the average elapsed time per question and the NASA-TLX score are negatively correlated (−.72 and −.66, respectively) to participants’ performance and significant (*P*=.02 and *P*=.04, respectively). For the graphic-based task, the average response time, average elapsed time per question, and NASA-TLX score are negatively correlated (−.78, −.93, and −.79, respectively) to the participants’ performance and significant (*P*=.008, *P*<.001, and *P*=.006, respectively).

**Table 4:** Correlation analysis for the text- and graphic-based tasks.

Measure		Correlation value	*P*
**Text-based task**			
	Average response time	−.84	.002
	Average elapsed time per question	−.72	.02
	NASA-TLX^a^	−.66	.04
**Graphic-based task**			
	Average response time	−.78	.008
	Average elapsed time per question	−.93	<.001
	NASA-TLX^a^	−.79	.006

^a^National Aeronautics and Space Administration task load index

## Discussion

### Principal Findings

Our study showed that patients with early stages AD could use mobile devices successfully without any prior experience. Participants were able to comprehend, recognize, and recall information from our mobile app ADcope, and performed better in the graphic-based tasks compared to text-based tasks with a lower response time. The workload for completing these tasks was low, and participants were satisfied in completing the tasks successfully based on the posttask questionnaire scores. These are good indications for the app, as Yurko et al [9] showed that increased workload during task performance may increase fatigue and facilitate errors, which could affect the app’s overall effectiveness.

### Comparison With Prior Work

Many researchers have focused on using mobile devices and social networks for monitoring elderly patients by relatives and health professionals [10-12]. Chan et al [13] investigated the use of smart homes for elderly where intelligent devices including sensors and assistive robotics are implanted into their homes for continuous mobility assistance and nonobtrusive disease prevention.

Some researchers proposed various devices to help AD patients to cope with the symptoms. Imbeault et al [14] proposed using an electronic organizer to help individuals with AD to organize their daily activities. Tapus [15] suggested using socially assistive robotic system that aims to provide a customized protocol through motivation, encouragement, and companionship for users suffering from cognitive changes related to aging and/or AD.

Bouchard et al [16] proposed guidelines for designing and implementing effective serious games targeting Alzheimer’s patients. The suggested guidelines include designing appropriate interaction mechanisms for cognitively impaired people, implementing artificial intelligence for providing adequate assistive prompting and dynamic difficulty adjustments, and producing effective visual and auditory assets to maximize cognitive training.

Lim et al [17] explored the usability of tablet computers within the early-stage dementia context as a source of leisure for people with dementia. The authors’ results indicated that one-half the participants with dementia were able to engage with and use the tablet computer independently, which proved to be helpful to their caretakers.

Much research has focused on usability studies of devices, apps, and games that are targeting Alzheimer’s patients. Boulay et al [18] conducted a usability study of MINWii, a music therapy game for patient suffering from mild to moderately severe AD. The authors demonstrated that patients were satisfied with the game and expressed a desire to repeat the experience. Granata et al [19] completed a usability testing of the graphical user interface for robot service for elderly with cognitive impairment. The authors concluded despite that cognitive profile, age, and computer experience were found to impact task performance, the interfaces and contents of the services assessed were usable by older adults with cognitive impairment. Nugent et al [20] carried a usability study for mobile phone–based video reminding system along with a qualitative collection of pre- and postevaluation questionnaires. The authors suggested that caretakers played a significant role in terms of the success of the solution and that an initial settling was required before users felt comfortable using the technology.

### Conclusions

AD is the most common cause of dementia among older people causing the loss of cognitive functioning including thinking, remembering, and reasoning. Currently, there is no cure for the disease, which worsens as it progresses, and eventually leads to death. In this paper, we have conducted usability study for the spaced retrieval memory exercise using mobile devices on 10 participants with early stages of AD. We have found that people with early stages of AD have been successfully able to use the mobile device without having any prior experience in using such devices. Participants’ measured workload was found to be low and posttask satisfaction in fulfilling the required task has shown to be conceivable. Results also indicated better performance, less workload, and better response time for the graphic-based user interface compared with the text-based user interface.

## Abbreviations

AD: Alzheimer’s disease
ANOVA: analysis of variance
NASA-TLX: National Aeronautics and Space Administration task load index
NFC: near field communication
OS: operating system
SDK: software development kit
